# Vascular Expression of Hemoglobin Alpha in Antarctic Icefish Supports Iron Limitation as Novel Evolutionary Driver

**DOI:** 10.3389/fphys.2019.01389

**Published:** 2019-11-12

**Authors:** Bruce A. Corliss, Leon J. Delalio, T. C. Stevenson Keller, Alexander S. Keller, Douglas A. Keller, Bruce H. Corliss, Jody M. Beers, Shayn M. Peirce, Brant E. Isakson

**Affiliations:** ^1^Biomedical Engineering Department, University of Virginia, Charlottesville, VA, United States; ^2^Robert M. Berne Cardiovascular Research Center, University of Virginia School of Medicine, Charlottesville, VA, United States; ^3^Department of Molecular Physiology and Biophysics, University of Virginia School of Medicine, Charlottesville, VA, United States; ^4^Sanofi, Paris, France; ^5^Graduate School of Oceanography, University of Rhode Island, Kingston, RI, United States; ^6^Department of Biology, College of Charleston, Charleston, SC, United States

**Keywords:** hemoglobin alpha, evolution, vasculature, iron flux, endothelial cells

## Abstract

Frigid temperatures of the Southern Ocean are known to be an evolutionary driver in Antarctic fish. For example, many fish have reduced red blood cell (RBC) concentration to minimize vascular resistance. *Via* the oxygen-carrying protein hemoglobin, RBCs contain the vast majority of the body’s iron, which is known to be a limiting nutrient in marine ecosystems. Since lower RBC levels also lead to reduced iron requirements, we hypothesize that low iron availability was an additional evolutionary driver of Antarctic fish speciation. Antarctic Icefish of the family *Channichthyidae* are known to have an extreme alteration of iron metabolism due to loss of RBCs and two iron-binding proteins, hemoglobin and myoglobin. Loss of hemoglobin is considered a maladaptive trait allowed by relaxation of predator selection since extreme adaptations are required to compensate for the loss of oxygen-carrying capacity. However, iron dependency minimization may have driven hemoglobin loss instead of a random evolutionary event. Given the variety of functions that hemoglobin serves in the endothelium, we suspected the protein corresponding to the 3’ truncated Hbα fragment (Hbα-3’f) that was not genetically excluded by icefish may still be expressed as a protein. Using whole mount confocal microscopy, we show that Hbα-3’f is expressed in the vascular endothelium of icefish retina, suggesting this Hbα fragment may still serve an important role in the endothelium. These observations support a novel hypothesis that iron minimization could have influenced icefish speciation with the loss of the iron-binding portion of Hbα in Hbα-3’f, as well as hemoglobin β and myoglobin.

## Introduction

The waters of the Southern Ocean are the coldest on Earth, with temperatures beneath the surface varying +1.5°C (Sidell and O’Brien, 2006) to as low as −2.13°C (Jacobs et al., 1970). Such frigid conditions are lethal for most mammalian species where blood plasma will quickly freeze (Verde et al., 2007), yet this marine environment hosts many fish species and mammals in an ecosystem that exhibits an unexpectedly high amount of biomass despite the harsh conditions (Lam and Bishop, 2007; Thresher et al., 2011). Species of the suborder Notothenioidei make up 90% of the fish biomass in the seas surrounding Antarctica (Sidell and O’Brien, 2006), and to withstand cold conditions, they have evolved adaptations such as decreased concentration of red blood cells to minimize blood viscosity. Previous research revealed that hematocrit closely correlated with temperature tolerance across species (Beers and Sidell, 2011). An underappreciated consequence of reduced hematocrit is a significant decrease in utilization of elemental iron, since 70% of red-blooded mammals’ iron is found in red blood cells in the oxygen-carrying protein hemoglobin (Andrews, 2000). Iron is not only considered an essential nutrient for use in various iron-binding proteins (Cairo et al., 2006), but is also established as a limiting nutrient in many aquatic ecosystems (Martin and Fitzwater, 1988) including the Southern Ocean (Thomas, 2003). Previous research indicates that addition of iron is sufficient to induce transient spikes in algal biomass (Buesseler et al., 2004). Bioavailable iron is primarily released from ground bedrock during periods of high glaciation, contrasted with up to a 20-fold reduction of iron concentration during periods of low glacial activity (Shoenfelt et al., 2018). Iron limitation is likely to have coincided with both of the theorized geologic eras for speciation of Antarctic icefish: compatible with formation of the clade approximately 15–20 million years ago prior to global cooling and glaciation of the Southern Ocean (Kock, 2005a), or more recently approximately 6 million years ago coinciding with one of many periods in between glaciation cycles (Martínez-Garcia et al., 2011). Therefore, we propose that iron limitation could be a selection pressure resulting in adaptations associated with iron binding proteins in the oceans surrounding Antarctica.

Out of all the Notothenioids, Antarctic Icefish of the family Channichthyidae are canonically known for the most extreme alterations in iron requirement, lacking expression of hemoglobin, and in half of all species, also myoglobin (Kock, 2005a). In this family of icefish, oxygenation is thought to occur purely through diffusion-based transport of dissolved oxygen in the blood (Sidell and O’Brien, 2006). The high energetic cost of circulating blood at a rate sufficient for diffusion-based oxygen transport has led previous research to conclude that hemoglobin loss is a net-negative or neutral trait that evolved by chance and remained due to relaxed predator selection (Sidell and O’Brien, 2006). However, preservation of such a deleterious trait, even when paired with relaxed selection pressure, is not consistent with the extreme cardiovascular adaptations found in icefish that are required to compensate for such inefficient diffusion-based oxygen transport (Kock, 2005a). Rather than the random loss of a beneficial trait followed by selection for several necessary compensatory traits, it is plausible that these traits are the product of directional selection resulting from an environmental factor such as limited iron availability.

Knock-out of hemoglobin occurred with the complete genomic deletion of hemoglobin beta (Hbβ), and partial ablation of hemoglobin alpha (Hbα). Previously, we have found evidence in vertebrates that Hbα modulates vascular remodeling through endothelial nitric oxide signaling (Straub et al., 2012; Lechauve et al., 2018), independent of its canonical function in blood oxygen transport. We hypothesized that this vascular-specific function could be preserved with the 3′ fragment of hemoglobin alpha (Hbα-3’f) found across the genomes of all icefish (Near et al., 2006) and previously classified as an inactive pseudo-gene (Cocca et al., 1995; Zhao et al., 1998; Near et al., 2006). We present evidence that Hbα expression in the endothelium has been preserved in icefish retina, a tissue that allows for ready visualization of the vasculature. However, structural analysis reveals that all known binding locations are excluded from the fragment, suggesting that this gene fragment may reveal an additional undiscovered role of Hbα in vascular remodeling. This result also prompts a reconsideration of whether Antarctic icefish are truly a complete hemoglobin knockout, and reveals the limitation of iron as a possible novel selection pressure in aquatic Antarctic environments. Studying hemoglobin expression in Antarctic icefish may yield insights into how icefish avoid pathological consequences from heightened endothelial nitric oxide production seen in other species and could inform future therapeutics modulating this fundamental vascular signaling pathway.

## Materials and Methods

### Animal Collection and Sample Preparation

Two species of Antarctic notothenioid fishes were collected from the waters of the Antarctic Peninsula region during the austral autumn (April–May) of 2009. *Champsocephalus gunnari*, an icefish species, was caught by otter trawls deployed from the ARSV *Laurence M. Gould* (expedition number LMG09-05) at water depth of 75–150 m in Dallmann Bay (64°08′S, 62°40′W). *Lepidonotothen squamifrons*, a red-blooded notothenioid, was collected in baited traps set at a depth of 200–500 m in both Dallmann Bay and Palmer Basin (64°50′S, 64°04′W). Animals were transferred to the US Antarctic research base Palmer Station, where they were maintained in flowing-seawater aquaria at ambient water temperatures of 0 ± 0.5°C. Individuals were first anesthetized in MS-222 in seawater (1:7, 500 w/v), and then killed by cervical transection. Retinal tissues were excised quickly, frozen in liquid nitrogen, and stored at −80°C until use as described in (Beers and Sidell, 2011) and originally by (Eastman, 1988). All research was in compliance with the University of Alaska guidelines for work conducted on vertebrate animals (institutional approval 134774-2) and endorsed by the University of Maine (UM) Institutional Animal Care and Use Committee. Fish collection was in accordance with the Antarctic Conservation Act of 1978 (ACA 2009-011).

### Whole Mount and Immunostaining

Retinal samples were thawed from storage at −80°C for 30 min or until equilibrium reached with room temperature. Samples were placed in a petri dish, freezing medium drained, and incubated in 4% PFA for 40 min, and washed three times with PBS for 5 min each. Tissue was then flat mounted on a microscope slide outlined with a hydrophobic pen (Sigma-Aldrich Z377821). For staining, samples were blocked and permeabilized with 1 mg/ml Digitonin (Sigma-Aldrich D141) with 10% normal donkey serum (Jackson ImmunoResearch Laboratories 017-000-121) for 3 h. The following primary antibodies were applied in Digitonin and samples incubated overnight: rabbit anti-hemoglobin beta (1:400, Abcam ab102758) as a negative control for no detectable expression, rabbit anti-hemoglobin alpha to detect Hbα-3’f (1:200, Abcam cat 102758), along with rat anti-CD31 (1:300, Biolegend 102504) and IB4 Lectin conjugated to Alexa Flour 488 (1:200, ThermoFisher 121411) to label the vasculature. Samples were washed with 0.2% saponin in PBS (Sigma-Aldrich S7900) and incubated overnight with the follow secondary antibodies in 1 mg/ml Digitonin: Donkey anti-rabbit (1:500, Abcam ab150155), Donkey anti-rat, DAPI (1:200, ThermoFisher D1306). Samples were then washed in 0.2% saponin in PBS six times for 30 min for 2 days and then mounted with a coverslip, sealed with nail polish. A single specimen of *Champsocephalus gunnari* and *Lepidonotothen squamifrons* was used for the immunostaining.

### Imaging

Samples were imaged on a laser point-scanning confocal microscope (Nikon Eclipse TE2000-E Confocal). Z stacks were acquired with a 20×/0.6 oil lens, using a 488 nm laser paired with a 515/30 bandpass, a 546 nm laser with a 590/50 bandpass, and 647 nm laser with a 650 long pass filter. Fluorophores were excited and imaged sequentially with each laser and filter combination to minimize crosstalk with 1,024 pixel resolution and saved as 8-bit images.

### Structure Visualization

Protein structure visualization of human deoxyhemoglobin was adapted from PDBid: 2HHB, and images created using PyMOL. The protein is rendered as a cartoon, and the 3’f colored blue using the graphics options in PyMOL. The antibody epitope is rendered as sticks based on manufacturer’s epitope data. The antibody captures a sequence that should be expressed by the Hba 3’f.

### Sequence Alignment and Phylogram

Protein sequences were obtained from Uniprot and NCBI model organism databases and aligned in the EMBL-EBI Clustal Omega alignment webserver.

Protein alignment for Hba-a1 gene was generated using the progressive alignment algorithm in the CLC Main Workbench software V8.0 (Qiagen^®^). Sequences were gathered from NCBI model organism database. Sequence alignment was accomplished using, no fix points, and cheap end gaps, and Gap open cost greater than Gap extension cost.

For the phylogram, branch location and length was calculated using neighbor joining and a Jukes-Cantor substitution model.

## Results

### Vascular Network and Hbα Fragment Localization *via* Whole Mount

**Figure 1:**
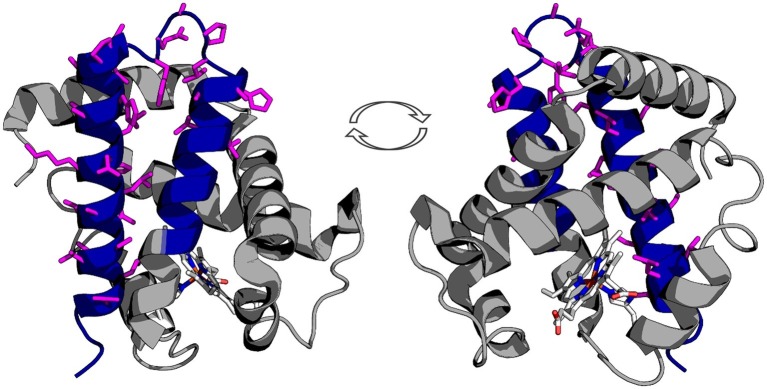
Representation of the truncated icefish alpha globin protein (blue) imposed on human deoxyhemoglobin (gray). The epitope for the antibody used in immunofluorescence is represented by magenta side chains, illustrating that the entire epitope lies within the truncated gene region. The prosthetic heme group is shown in elemental color scheme. Truncation of the hemoglobin in icefish removes residues critical for heme group stabilization and O_2_ binding ability. The views are a 180° rotation of the alpha globin molecule. Using PDBid: 2HHB.

First we mapped the predicted Hbα-3’f onto the hemoglobin alpha protein (blue; Figure 1) and identified topographically the protein was lacking the heme-binding region. Next, we mapped an alpha globin antibody on the predicted Hbα-3’f (magenta; Figure 1).

**Figure 2:**
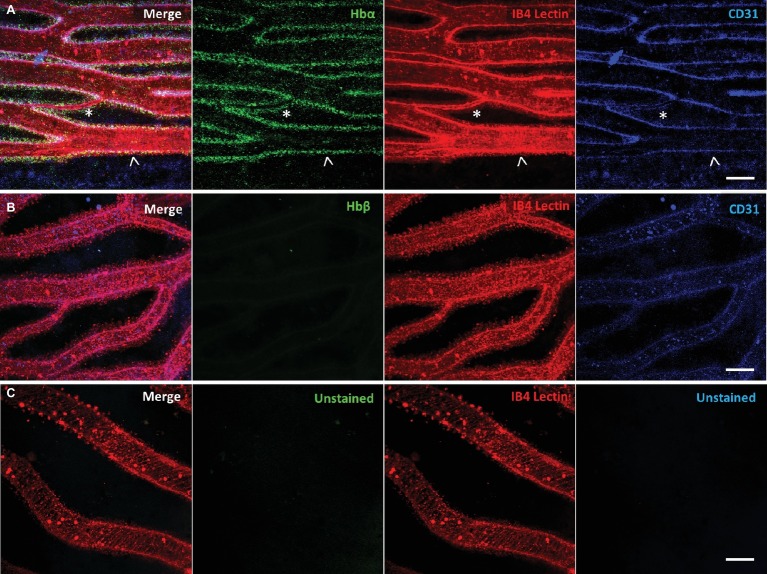
Icefish (*Champsocephalus gunnari*) hyaloid endothelial cells express hemoglobin alpha, while lacking hemoglobin beta expression. **(A)** Retinal surface labeled with anti-Hbα (green), IB4 lectin (red), and anti-CD31 (blue), with a network comprised of both large (arrow) and small (star) vessels. **(B)** Retinal surface labeled with anti-Hbβ (green), IB4 lectin (red) and anti-CD31 (blue). **(C)** Retina with lectin as stain control for other channels. Scale bar 100 μm, images acquired with 20×/0.75 objective (*N* = 1 fish immunostained).

Using our antibody against the Hbα-3’f, we investigated where Hbα protein was localized in whole-mount retinal tissue. In the hyaloid vessels of the vitreoretinal interface of *C. gunnari*, Hbα expression was localized within the vessel wall, denoted by CD31 and IB4 lectin, in blood vessels of all sizes (Figure 2A). As a negative control, there was no detectable expression of Hbβ as expected with its ablation from the icefish genome (Near et al., 2006) (Figure 2B), and no comparable signal observed in unstained tissue with only the secondary antibody present (Figure 2C).

**Figure 3:**
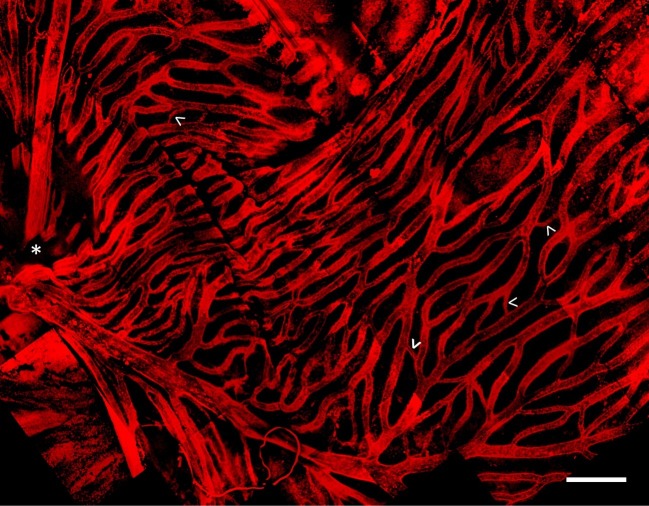
Hyaloid vascular network in vitreoretinal interface of icefish (*Champsocephalus gunnari*). Vasculature labeled with IB4 lectin (red), with thick vessels radiating from the optic disk (star, below imaging plane), connected with thin lateral vessels (arrow). Scale bar 1 mm, Images acquired with a 20×/0.5 objective (*N* = 1 fish immunostained).

Whole mount and immunostaining of *C. gunnari* retina revealed a dense network of IB4 lectin labeled hyaloid blood vessels in the vitroretinal interface radiating from a central optic disk (Figure 3). Of note were the large luminal diameters of the vessel network, with the smallest capillary diameter approximately 30 μm and the diameters of the primary vessels ranging from 30 to 150 μm, features that were lost in previous studies with lower resolution imaging modalities.

**Figure 4:**
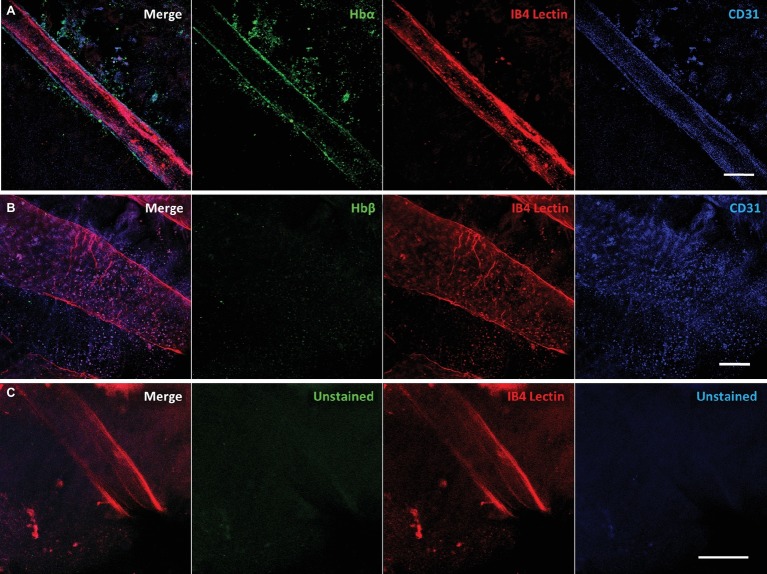
Red-blooded notothenioid (*Lepidonotothen squamifrons*) hyaloid endothelial cells express hemoglobin alpha, while lacking expression in hemoglobin beta. **(A)** Retinal surface labeled with anti-Hbα (green), IB4 lectin (red), and anti-CD31 (blue). **(B)** Retinal surface labeled with anti-Hbβ (green), IB4 lectin (red) and anti-CD31 (blue). **(C)** Retina labeled with lectin as stain control for other channels. Scale bar 100 μm, images acquired with 20×/0.75 objective (*N* = 1 fish immunostained).

To confirm that Hbα expression is present in more than one notothenioid, the retina of *Lepidonotothen squamifrons* was also examined. Whole mount immunostaining revealed Hbα expression localized to the endothelial cells contained within the vessel wall of the vasculature residing at the vitroretinal interface (Figure 4A). Similar to *C. gunnari*, there was no detectable Hbβ expression in *L. squamifrons* (Figure 4B), and no comparable signal was observed in unstained tissue with only the secondary antibody present (Figure 4C).

## Discussion

Using high-resolution confocal microscopy, our data demonstrate that fish devoid of RBCs, myoglobin, and hemoglobin β express the alpha hemoglobin fragment (Hbα-3’f) in their endothelium. This result demonstrates that alpha hemoglobin localization to endothelium is not confined to mammalian species, and more importantly, it may have more broad implications that originally thought. Some of these concepts are discussed below.

The frigid temperatures of Antarctica have contributed to numerous adaptations for organism to survive in low temperatures (Whittow, 1987; Cossins and Macdonald, 1989). The Southern Ocean is an especially unique environment because frigid temperatures lead to an oxygen-rich environment with near maximal oxygen saturation (Kock, 2005b). This unique combination of extreme environment conditions, paired with relaxation of selection from predation from a historical lack of apex predators in the food chain (Cocca et al., 1995), has led to especially extensive and unique temperature adaptations compared to more temperate locations.

Adaptations in the fish of the suborder Notothenioid, which make up 90% of the biomass in the Southern Ocean, include the formation of antifreeze glycoproteins that confer resistance to freezing (Coppes Petricorena and Somero, 2007) and enzymes optimized for activity at low temperatures to maintain metabolism in frigid conditions (Coppes Petricorena and Somero, 2007). These adaptations are mirrored by selection against traits for heat tolerance, such as the loss of functional heat-shock response genes found in other fish species that allow survival at warmer temperatures (Coppes Petricorena and Somero, 2007). These highly specialized adaptations to cold come at a cost, however, in that they make notothenioids highly stenothermal, able to survive only in a narrow temperature range from approximately −1.86 to 4°C (Ostadal and Dhalla, 2012).

Antarctic icefish of the family Channichthyidae are even more stenothermal than the other members of the suborder Notothenioidei (Cheng and William Detrich, 2007; Mueller et al., 2011, 11), with noticeable stress to the organism outside of the −2 to 2°C range (Sidell and O’Brien, 2006). These species are unique in the animal kingdom, exhibiting complete loss of red blood cells and Hbβ, as well as nearly complete loss of Hbα (Sidell and O’Brien, 2006). Yet the Channichthyidae family inhabits much of the same aquatic environment as the other notothenioids, typically between 800 and 1,500 m depth below sea level (Kock, 2005b). Since they coinhabit the same environment, comparing the evolution and physiology of icefish to those of closely related red-blooded notothenioids may yield insight into their diversification. This examination may reveal whether other evolutionary factors, such as minimization of the limiting nutrient iron, played a role in the unique adaptations found in these fish and other species of the Southern Ocean.

### Iron Flux Hypothesis: Iron Limitation as a Notothenioid Evolutionary Driver

Similar to the hemoglobin loss found in channichthyids, other notothenioids have evolved a low red blood cell count to counteract the approximately 40% increase of blood viscosity as salt water temperatures near freezing (Near et al., 2006). Hematocrit correlates robustly with thermal tolerance across species (Beers and Sidell, 2011). The loss of oxygen carrying capacity that results from reduced hemoglobin expression is viable in cold water environments because oxygen saturation in saltwater nears maximal levels as temperature approaches freezing (Mel’nichenko et al., 2008). Since hemoglobin (with bound iron) makes up 90% of the dry weight content of red blood cells (Rishi and Subramaniam, 2017), iron levels in an organism correlate with hematocrit. Hematocrit levels for many of the Notothenioids species are often below 25% (Beers and Sidell, 2011), with Antarctic icefish lacking hematocrit entirely, compared to 40–50% typically found in humans.

Iron is critical for several basic biologic functions, including cellular aerobic respiration, oxygen transport through the circulatory system *via* hemoglobin, and myoglobin function in skeletal muscle (Kaplan and Ward, 2013). In humans and red-blooded vertebrates, approximately 70% of the body’s iron content is found in hemoglobin in red blood cells (Andrews, 2000), 15% in myoglobin in muscle tissue (Kaplan and Ward, 2013), 6% in other proteins essential for cell metabolism, neurotransmission, and immune system function, and the remaining 9% kept in reserve. Considering the additional storage and trafficking requirements needed to supply iron for higher hematocrit (Gammella et al., 2014), Notothenioids could have a reduction in iron demand approaching 50% of that needed by many temperate fish species (Gallaugher and Farrell, 1998).

In oceanography, the Iron Hypothesis posits that iron is a limiting nutrient in oceanic ecosystems, sufficient to produce phytoplankton blooms on a large scale (Martin et al., 1994). Iron has been demonstrated as a limiting nutrient for biomass in a multitude of open ocean experiments, including the Southern Ocean (Conway et al., 2015). Polar oceans are especially known to have deficiencies in iron content and flux (Street and Paytan, 2005), resulting from the limited input from benthic sediment, atmospheric deposition, and icebergs, alongside limited trafficking of iron between vertical layers of ocean waters (Graham et al., 2015). There have been previous documented cases of limiting nutrients serving as a driver for evolution with plants (Lynch and Brown, 2001; López-Bucio et al., 2003; Rennenberg and Schmidt, 2010) and microorganisms (Lewis et al., 1986; Merchant and Helmann, 2012), providing ample precedent for the possibility that iron limitation may be an underappreciated driver of Antarctic aquatic speciation. Mammals have all developed highly specialized iron-binding proteins that act as means of transport and storage (Ganz and Nemeth, 2012), offering further demonstration that iron availability can be an significant evolutionary driver. Such extensive biological machinery is necessary because iron is an essential nutrient for all vertebrates (Chen and Paw, 2012), and while plentiful on the earth’s surface, is found in low amounts in bioavailable forms (Monsen, 1988). Additionally, atomic iron must be kept bound to proteins in a chaperoned state because free iron induces free radical formation that can damage tissue (Emerit et al., 2001).

Further evidence of iron minimization adaptations include Southern Ocean phytoplankton, autotrophs that form the base of the aquatic food chain (D’Alelio et al., 2016), that exhibit unique adaptations that reduce biochemical demand and increase the intracellular flux of bioavailable forms of iron (Strzepek et al., 2011), leading to an 80% reduction in iron requirements compared to temperate oceanic species (Lane et al., 2009). This scarcity of iron availability at the bottom of the food chain means that organisms higher on the food chain only receive a fraction of the iron per mass from phytoplankton compared to other environments, hinting at the Southern Oceans’ unique iron flux. Minimization of iron requirements across the food chain could allow an ecosystem to support more biomass than otherwise possible. Despite the harsh conditions, there is indeed evidence of higher total biomass than expected in the Southern Ocean (Lam and Bishop, 2007; Thresher et al., 2011). Comparing biomass production and iron flux between Antarctic and temperate aquatic environments with ecosystem-level modeling awaits confirmation, but may provide insight to unique iron utilization efficiency between them. Previous studies along these lines have revealed that the evolution of crustaceans in Antarctic seas were heavily influenced by an abundance of Magnesium, another element found in key signaling pathways (Frederich et al., 2000, 2001). This research lays precedence that an abundance of an element can significantly alter adaptations in aquatic polar environments.

We posit that limited iron availability in aquatic Antarctic environments has led to the selection of traits that conserve its use. In warmer aquatic environments, reducing hemoglobin iron content comes at a steep cost of oxygen-carrying capacity, aerobic respiration ability, and overall organismal fitness. In frigid environments, however, organisms that minimize red blood cell count and iron content would theoretically have the dual benefits of decreased dependence on iron for biomass support paired with an added benefit of lower blood viscosity from reduced hematocrit. Therefore, the oxygen-rich cold waters surrounding Antarctica are uniquely positioned to encourage decreased independence on iron *via* tenable trade-offs for organismal survival and species fitness. We propose that iron limitation could be a significant driver of icefish evolution, and possibly of portions of the Antarctic ecosystem as a whole.

### Antarctic Icefish as Model of Extreme Iron Metabolism Adaptations

Icefish from the family *Channichthyidae* are known for especially extreme alterations in iron metabolism, making their phylogenic history ideal for examining the evolutionary drivers related to iron minimization. Analysis of iron metabolism in icefish reveals an organism optimized for low iron requirements. Although we present evidence of the expression of a truncated Hbα fragment in icefish tissue, the iron-binding portion of the protein has been ablated along with the entire Hbβ reading frame in all but one icefish species, with *Neopagetopsis ionah* retaining both hemoglobin subunits but thought to form a nonfunctioning complex (Cocca et al., 1995; Near et al., 2006). Loss of hemoglobin and red blood cells leads to a 90% decrease in oxygen-carrying capacity (Wujcik et al., 2007) and up to 40% decrease in blood viscosity (Sidell and O’Brien, 2006) compared to red-blooded notothenioids. Oxygen transport is therefore purely driven from passive diffusion of surrounding blood vessels into peripheral tissues, dramatically reducing the ability of the circulatory system to deliver sufficient oxygen (Garofalo et al., 2009b). Not only is the iron demand from hemoglobin absent in icefish, but 6 of the 16 species of Antarctic icefishes have also lost myoglobin expression, an iron binding protein found in muscle tissue and used for oxygen storage (Sidell and O’Brien, 2006). Intriguingly, previous research has concluded that this myoglobin loss was carried out *via* four independent events during radiation of the species (Sidell and O’Brien, 2006), illustrating what could be a strong diversifying selection pressure on icefish myoglobin expression.

Based on iron distribution of red-blooded vertebrates, exclusion of hemoglobin and myoglobin in an organism could incur a 90% reduction in iron demands required for homeostasis. Indeed, without iron-binding hemoglobin, iron content in icefish blood plasma is less than 5% of closely related red-blooded species (di Prisco et al., 2002). Yet there is even further evidence of additional iron minimization beyond loss of hemoglobin and myoglobin: concentrations of non-heme iron in Antarctic icefish plasma are one-sixth of that in closely related red-blood species, and are lower by roughly half across various tissues (Kuhn et al., 2016). With a tissue level reduction in iron content, paired with the knockout of two primary iron binding proteins, the iron requirements of icefish normalized to biomass could be greater than 95% compared to other organisms and awaits confirmation.

### Iron Minimization Explains Antarctic Icefish Hemoglobin Loss

The loss of hemoglobin is thought to be a non-beneficial evolutionary event paired with a series of compensatory vascular adaptations meant to counteract the loss of oxygen-carrying capacity (Kock, 2005a). An energetic analysis of icefish suggests that cardiac function accounts for 22% of resting metabolic demand in icefishes, compared to around 3% with other notothenioids (Sidell and O’Brien, 2006). Consequently, hemoglobin loss is perceived as an energetic net negative, requiring far more energy for circulating the high volume of blood plasma required for sufficient oxygen transport than with hemoglobin-mediated oxygen transport (Sidell and O’Brien, 2006). Hemoglobin loss is seen as an evolutionary accident, hypothesized to be caused by the presence of a recombination hotspot within the hemoglobin reading frame (Cheng and William Detrich, 2007). This predisposition of the disruption of the hemoglobin gene complex (Cocca et al., 1995), paired with a relaxation of selection pressure from predators and oxygen transport from colder temperatures during the speciation of icefish, allowed for the non-beneficial trait to be passed on (Cocca et al., 1995).

**Figure 5:**
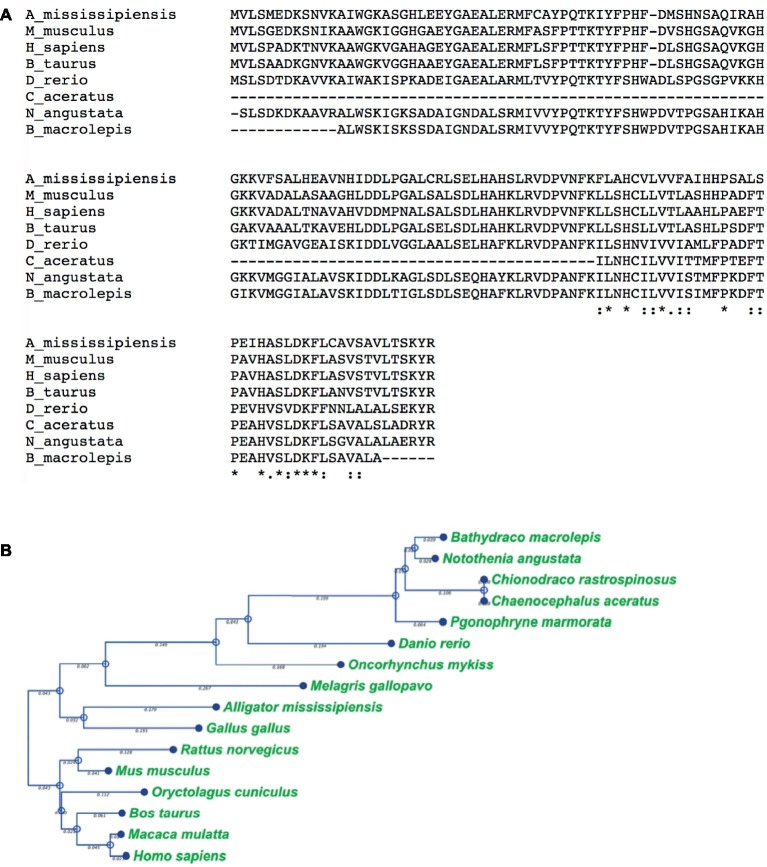
The truncated alpha globin found in icefish is similar to sequences found in other species of fish and the human sequence. **(A)**, Alignment of icefish, icefish-related fish, and human alpha globin sequences using Clustal Omega sequence alignment tool. Symbols “*,” “:,” and “.” represent degree of similarity between amino acids across the sequences. The truncated alpha globin found in icefish is similar to sequences found in other species of fish and the human sequence. C aceratus is the 3’f of the icefish in these sequences. **(B)**, phylogenic tree was constructed using distance-based Neighbor Joining method and protein distance measured with Jukes-Cantor distance adjustments Bars denote branch length for the amount of change between sequence nodes.

We provide evidence that a conserved fragment of Hbα is expressed in the vessel walls of the retina of an icefish species, providing evidence that the protein is translationally active. While previous research uniformly references the complete lack of hemoglobin expression in icefish (di Prisco et al., 2002; Kock, 2005a; Sidell and O’Brien, 2006; Cheng and William Detrich, 2007; Mueller et al., 2011), Hbα expression has only been examined in a single species, with mRNA probed indirectly *via* southern blot with Hbα cDNA fragments from a related red-blooded species (Cocca et al., 1995). Intriguingly, the protein fragment that is detected excludes known interaction and coordination sites, lacking known binding sites for heme [from Leu(F1) to Phe(G5) (Inaba et al., 1998)], eNOS [amino acid sequence LSFPTTKTYF (Keller et al., 2016)], and the α-hemoglobin stabilizing protein that inhibits Hbα precipitation (Figure 1; Feng et al., 2004). In addition to the endothelial-specific promoter machinery preserved in icefish, BLAST analysis of the Hbα fragment demonstrates high homology with red-blooded vertebrates and humans (Figure 5A), and the fragment has been conserved with the species and other vertebrates throughout the phylogenic tree (Figure 5B).

The preservation of functional endothelial-specific expression and conservation of amino acid sequence despite ablation of the majority of the gene suggests that a selection pressure has prevented the complete loss of Hbα. This resistance to complete ablation is most likely explained by Hbα-3’f expression significantly contributing to the fitness of the organism, with complete loss or variation in amino acid sequence being detrimental or even lethal. When considered with the genomic ablation of related iron-binding genes Hbβ and myoglobin, the extreme alterations of three iron binding genes makes it unlikely that these changes are a result of random genetic drift, but implies instead that there was some selection pressure. These changes suggest that rather than a maladaptive (Near et al., 2006) or coincidental neutral benefit trait (Sidell and O’Brien, 2006), loss of Hbα could have been the product of diversifying selection pressure, favoring a range of hemoglobin phenotypes (Bargelloni et al., 1998) driven by the recombination hotspot found in close proximity in the genome to Hbα (Cheng and William Detrich, 2007), with a niche favoring the near complete loss of the gene.

The existence of an evolutionary driver for hemoglobin loss is further supported by the extreme vascular adaptations required to compensate for the loss of hemoglobin-mediated oxygen carrying capacity. Antarctic icefish exhibit dramatic cardiac hypertrophy (Doake, 1987) and a 6-15 fold increase in pump volume compared to other teleosts (Hemmingsen et al., 1972), leading to a dramatic increase in cardiac output (Garofalo et al., 2009b). Thin, scaleless skin facilitates cutaneous oxygen absorption (Kock, 2005a), although its contribution to total oxygen supply is thought to be minor (Doake, 1987). *Via* higher blood vessel density and larger capillary diameter (Wujcik et al., 2007), the icefish vasculature contains 4-fold greater blood volume than red-blooded notothenioids (Garofalo et al., 2009b), resulting in higher oxygen flux to compensate for the reduced oxygen carrying capacity of diffusion-based oxygen delivery. To minimize oxygen demand, icefish have also evolved lower metabolism (O’Brien et al., 2003; Kock, 2005a) and enhanced mitochondrial biogenesis (Coppe et al., 2013).

Building upon prior findings, our results indicate a unique vascular structure of hyaloid vessels at the vitreoretinal interface in the icefish species *C. gunnari*. Icefish retinae have been previously visualized on a macroscopic scale through perfusion of opaque silicon rubber and imaged with light photography (Wujcik et al., 2007). Those images revealed a dense hyaloid vascular network branching out from a central optic disk connected to a dense and high-volume capillary network composed of highly isolated branches with few cross-connecting vessels. Higher resolution confocal images of immunostained *C. gunnari* retina reveal a similar basic vascular network structure, but also a prevalence of smaller vessels connecting vessel branches to form a highly interconnected vessel network. Vessels ranged from 30 μm for the smallest connecting capillaries to 150 μm for the primary vessels emerging from the optic disk. The unusual thickness of these vessels compared to those typically found in vertebrates (Egginton et al., 2002) corroborates previous research demonstrating that mean capillary diameters in Antarctic icefish are 50% larger than capillary diameters in the retina (Wujcik et al., 2007) and skeletal muscles (Egginton et al., 2002) of red-blooded notothenioids. The high density and thickness of the retinal capillary bed is attributed to adaptation to the cold environment of the Antarctic waters (Cheng and William Detrich, 2007) to minimize vasculature resistance and maximize oxygen diffusion. The energetic investment needed to maintain such a wide range of adaptations that are required to oxygenate tissues without hemoglobin-mediated oxygen transport further suggests that intense selection pressures are responsible for their initiation and preservation in the gene pool. While there is precedence of complex compensatory adaptations for traits seen as maladaptive, such as the evolution the mammalian retina with sight cells positioned on the far side of the tissue opposite incoming light (Dawkins, 1991), there are a lack of cases where a maladaptive trait associated with organism morbidity is retained through a series of compensatory adaptations (Crespi, 2000) as seen with icefish hemoglobin loss.

Icefishes co-inhabit the same environments as closely related red-blooded notothenioids, and there is no evidence of any advantages to fitness with hemoglobin loss (Sidell and O’Brien, 2006). In fact, evidence points to the reverse, where hemoglobin loss is paired with significant metabolic trade-offs compared to closely related red-blooded notothenioids (Sidell and O’Brien, 2006), casting doubt on the possibility of a pure directional selection pressure on hemoglobin concentration, where the extreme phenotype of hemoglobin loss yields a competitive advantage. If the near complete loss of hemoglobin is driven by a diversifying selection pressure, rather than a maladaptive event or neutral drift, then an additional driver could be required to fully explain why a diversity of phenotypes of hemoglobin concentration are found in fish of the Southern Ocean (Beers and Sidell, 2011). We propose that iron limitation may be the missing evolutionary force behind icefish adaptation, and an underappreciated driver with the evolution of many of Antarctic aquatic species in general. We present evidence that supports the notion that a diversifying selection pressure may have driven icefish evolution with hemoglobin-mediated oxygen transport, where frigid temperatures and minimization of blood plasma viscosity are insufficient to explain the driving forces behind icefish evolution. Minimization of iron requirements could contribute to organism fitness when prey populations are restricted or contain reduced iron content, as found with phytoplankton (Strzepek et al., 2011) and fish (Beers and Sidell, 2011) in the Southern Ocean. Low iron usage may have aided survival during the crash in Antarctic biodiversity that co-occurred with icefish speciation (Eastman, 1993) roughly 8.5 million years ago (Near, 2004). The evolutionary importance of conservation of metabolic inputs has precedence that includes adaptations with hibernation of mammals in winter (Geiser, 2013), dormant states in bacteria during environmental stress (Watson et al., 1998), and starvation responses found across mammals (Wang et al., 2006).

### Antarctic Icefish as a Model of Heightened Endothelial NO Bioavailability

Recently, hemoglobin alpha, canonically known for its role in oxygen transport *via* binding with hemoglobin beta in red blood cells, has been shown to modulate vascular remodeling in protrusions of endothelial cells called myoendothelial junctions (MEJ) (Straub et al., 2014). These regions are on the basolateral membranes of endothelial cells, proximal to smooth muscle cells, and facilitate communication between the two cell types in the vascular wall. Hbα modulates endothelial NO flux at the MEJ (Straub et al., 2012) in resistance arteries by binding to eNOS and acting as a scavenger of NO (Butcher et al., 2014). Due to NO’s short biological half-life (Thomas et al., 2001), Hbα serves as a significant negative regulator for the availability of endothelial NO reaching proximal smooth muscle cells. Disruption of the Hbα-eNOS interaction can lead to smooth muscle vasodilation and reduction in blood pressure (Keller et al., 2016), while eNOS inhibition leads to vasoconstriction of the peripheral vasculature and can induce significant increases in blood pressure (Li and Förstermann, 2000). Indeed, pharmacologically increasing the bioavailability of endothelial NO (Kurowska, 2002) is perceived as a promising therapeutic strategy in atherosclerosis (Barbato and Tzeng, 2004), ischemia (Barbato and Tzeng, 2004), diabetes (Masha et al., 2011), and hypertension (Hermann et al., 2006). Paradoxically, completely unregulated hyperactive endothelial NO generation can be pathological. A pronounced example is a recent preclinical study in rhesus monkeys, where an antibody exhibiting off-target effects leading to elevated NO production (Pai et al., 2016) caused severe systemic vasodilation, as well as hypotension, hematemesis, hematochezia, and morbidity. Additionally, elevated levels of NO is used a biomarker is various diseases (Arkenau et al., 2002; Pham et al., 2003), results in apoptosis (Blaise et al., 2005), produces cytotoxic oxygen radicals, exerts cytotoxic and antiplatelet effects (Sim, 2010), inhibits enzyme function, promotes DNA damage, and activates inflammatory processes (Hollenberg and Cinel, 2009).

In the absence of Hbα scavenging NO, as evident *via* the exclusion of the binding sites for NOS and heme in Hbα-3’f, production of NO might be up-regulated. Icefish could potentially serve as a model organism to study up-regulation of endothelial nitric oxide signaling (Beers and Jayasundara, 2015) while avoiding the pathological ramifications that are experienced in Hbα-expressing vertebrates (Pai et al., 2016). A possible function of Hbα-3’f could include NO binding at Cysteine 5 in a similar fashion to the established Cysteine-NO interaction found at Cysteine 93 in Hbβ (Sampath et al., 1994; Helms and Kim-Shapiro, 2013). Instead of trapping NO in the Hbα-eNOS complex at the point of generation, NO trapping would be carried on in a diffuse form with the freely disassociated Hbα fragment throughout the cytosol. Altered nitric oxide kinetics could represent a safe method to up regulate nitric oxide metabolites in the vessel wall while still maintaining negative regulation that avoids NO toxicity.

The vascular evolutionary adaptations compensating for loss of heme-mediated oxygen-carrying capacity are thought to be facilitated through nitric oxide signaling (Cheng and William Detrich, 2007). Enriched endothelial NO has been shown to play a role in modulation of vasodilation (Palmer et al., 1987), angiogenesis (Ziche and Morbidelli, 2000), cardiac hypertrophy (Wollert and Drexler, 2002), mitochondria size (Urschel and O’Brien, 2008), and mitochondrial biogenesis (Nisoli and Carruba, 2006; O’Brien and Mueller, 2010), all of which are exaggerated phenotypes found in hemoglobin-lacking icefish (Kock, 2005a). Several studies provide evidence of the presence of a functional NOS signaling system (Pellegrino et al., 2004) and expression of eNOS has been preserved in endothelial cells of icefishes (Garofalo et al., 2009a), along with a 50% greater plasma load of NO metabolites (NO_x_) in icefish compared to red-blooded notothenioids (Beers et al., 2010). However, it is important to note that a significant portion of this elevated NO metabolite load could be from a physiologic response to hemoglobin loss, revealed that, at least in a transient fashion, when red-blooded notothenioids were subject to chemically induced anemia that resulted in a 70-90% reduction in hemoglobin concentration, NOx metabolites also increased by 30% (Borley et al., 2010).

Nitric oxide metabolite buildup in icefish is theorized to be caused by reduced degradation rather than increased generation. Previous studies have shown that vascularized icefish tissue has a 50% decrease of NOS (Beers et al., 2010), the primary source of endothelial NO generation, compared to closely related red-blood species. The alteration of Hbα’s heme-based NO scavenging ability in the Hbα-3’f could explain how icefish simultaneously express less NOS but exhibit greater NO load in the vasculature (Beers et al., 2010).

## Conclusion

We provide evidence that Hbα-3’f is expressed transcriptionally, translationally, and localized to the vasculature in *C. gunnari*. Conservation of the Hbα-3’f amino acid sequence between icefish species and red-blooded vertebrates, along with preservation of endothelial-specific promoter machinery alongside loss of all known Hbα interaction regions, suggests that this Hbα-3’f fragment plays a novel, unknown role in the endothelium. These findings demonstrate that icefish do not technically have both hemoglobin genes knocked out, but do suggest that all known Hbα functions have been disabled, including known interaction regions with eNOS, heme, and NO. The ablation of the majority of the Hbα gene may essentially represent a natural mutagenesis experiment where nonlethal portions of the gene are eliminated, offering a possible opportunity to identify a novel role of the Hbα fragment region that may translate back to red-blooded vertebrates.

Preservation of the Hbα-3’f protein fragment suggests that a diversifying selection pressure could have driven the process. We propose that iron is a novel evolutionary driver for icefish hemoglobin loss, and perhaps even for the decreased hemoglobin concentration found in various other Antarctic aquatic species. Testing this hypothesis will require an examination of iron flux and iron utilization on an organism and ecosystem level.

## Data Availability Statement

All datasets generated for this study are available from the corresponding author on reasonable request.

## Ethics Statement

Ethical review and approval was not required for the animal study because the fish used in this research were caught in the ocean.

## Author Contributions

BAC performed experiments and wrote manuscript with input from all authors. LD performed NCBI sequence alignment and phylogenic analysis. TS conducted structural analysis. AK and DK aided with drafting of manuscript. BHC aided with drafting of manuscript and interpretation of results. JB collected specimens and tissue samples, aided with drafting of manuscript and interpretation of results. SP aided with drafting of the manuscript and interpretation of results. BI conceived the idea, aided with drafting of the manuscript, interpretation of results, and supervised the project.

### Conflict of Interest

DK was employed by the company Sanofi, Paris, France.

The remaining authors declare that the research was conducted in the absence of any commercial or financial relationships that could be construed as a potential conflict of interest.
